# Clinical effect of in-house rapid diagnostic process on patients with bloodstream infections due to carbapenem-resistant bacteria or methicillin-resistant *Staphylococcus aureus*: a prospective cohort study

**DOI:** 10.1128/spectrum.01746-25

**Published:** 2026-01-08

**Authors:** Hongwei Pan, Xiaoyu Zhang, Yiwen Gao, Wei Li, Zhaogang Dong, Zhengdong Luo, Yong Li, Xiaoli Zhang, Yue Wu, Hongxia Zhou, Ying Wang, Yue Sun, Yanyan Liu, Miaomiao Mi, Enhua Sun, Hao Wang, Yi Zhang

**Affiliations:** 1 Department of Clinical Laboratory, Qilu Hospital of Shandong University 91623, Jinan, Shandong, China; 2 Department of Hematology, Qilu Hospital of Shandong University 91623, Jinan, Shandong, China; 3 Shandong Engineering Research Center of Biomarker and Artificial Intelligence Application, Jinan, Shandong, China; 4 Clinical Molecular Diagnostics Institute of Shandong University, Jinan, Shandong, China; 5 Department of Critical Care Medicine, Qilu Hospital of Shandong University 91623, Jinan, Shandong, China; Inflammatix Inc., Sunnyvale, California, USA

**Keywords:** clinical effect, rapid diagnostic, bloodstream infections, CRO, MRSA

## Abstract

Bloodstream infections caused by multidrug-resistant bacteria result in a high mortality rate. Little research has been carried out to analyze the clinical effects of laboratory-based rapid identification and susceptibility tests of the types of organisms responsible for these infections. We previously developed an ammonium chloride-based in-house workflow for direct pathogen identification and susceptibility testing from positive blood cultures, eliminating the need for subsequent overnight subculturing. In this study, we carried out a prospective single-center cohort study evaluating the outcomes associated with in-house rapid detection of carbapenem-resistant bacterial strains or methicillin-resistant *Staphylococcus aureus* directly from positive blood cultures. From 2018 to 2023, 584 patients with clinically significant monomicrobial bloodstream infections due to these strains were enrolled. Among the 584 patients, 412 were included in the final analysis, with 182 in the in-house group and 230 in the conventional group. The median time from positive blood culture to identification and susceptibility results was significantly shorter for the in-house rapid process than for the conventional process (*P* < 0.01). A decrease in laboratory report time improved the time to effective antibiotic therapy and increased the adjustment rate of effective antibiotics from 56.09% (129/230) to 70.33% (128/182) (*P* < 0.01). The 28-day all-cause mortality after occurrence of a bloodstream infection markedly decreased from 19.57% (45/230) to 9.34% (17/182) (*P* = 0.005). Our results indicate that the in-house rapid process improves the time to effective antibiotic therapy and significantly decreases patient mortality. This information should encourage the implementation of our in-house rapid diagnostic process for bloodstream infections.

**IMPORTANCE:**

In this study, we included only patients whose bloodstream infection was caused by carbapenem-resistant bacterial strains or methicillin-resistant *Staphylococcus aureus*, avoiding the interference of empirical treatment for sensitive strains and allowing a clearer evaluation of the effects of laboratory-based rapid workflows. Moreover, this study covered all bloodstream infection cases from 1 January 2018 to 30 June 2023, with patients randomly grouped to minimize confounding factors. The results of this study demonstrated that this laboratory-based rapid workflow significantly shortened laboratory reporting times, which led to improvements in the rate and timing of adjustment of effective antibiotic therapy and a significant decrease in the mortality of patients with bloodstream infections. To the best of our knowledge, few studies have employed such a procedure to analyze clinical effects. Therefore, we believe that our research findings will encourage the implementation of laboratory-based rapid diagnosis for bloodstream infections.

## INTRODUCTION

Bloodstream infections (BSIs) and severe sepsis remain significant public health concerns. The administration of optimal and timely antibiotic therapy decreases the mortality of patients with BSIs (1). However, appropriate antibiotic prescribing can be challenging for clinicians, as the choice of antibiotic ultimately depends on identifying (ID) a causative pathogen and having antibiotic susceptibility test (AST) results. To decrease the mortality of patients with BSIs, empirical treatment is usually carried out before the causative pathogen ID and AST results are obtained. However, empirical treatment often fails to treat BSIs caused by carbapenem-resistant gram-negative bacterial strains (CRO) and methicillin-resistant *Staphylococcus aureus* (MRSA). All first-line clinical antibiotics are usually ineffective against these organisms, which is likely to lead to increased patient mortality (2, 3). Thus, we speculated that clinicians rely on early and accurate ID and AST results for these bacteria in blood cultures (BCs) to optimize therapy and decrease patient mortality.

Blood culture is the current standard approach for diagnosing BSIs (4). However, the conventional protocols for selecting the best specific treatment for bacteremia patients via AST are time-consuming. The entire process typically requires a turnaround time of at least 2–3 days. The long turnaround time is due mainly to the need for blood culture to amplify the number of pathogens, followed by the subculturing of positive cultures to obtain purified isolated colonies. This process might significantly delay the treatment of patients with BSIs, especially infections caused by CRO or MRSA. Recently, various novel technologies that aim to reduce the time to ID and AST have become available. Matrix-assisted laser desorption ionization time-of-flight (MALDI-TOF) mass spectrometry (5, 6), fluorescence *in situ* hybridization (FISH) (7–10), light scattering technology (11), FISH combined with time-lapse microscopy and nucleic acid amplification test-based methods (12), etc., have been developed for the rapid ID of pathogens causing BSIs. Adoption of these instruments can decrease the time needed to identify pathogens causing BSIs (9, 13, 14). Several published reports have evaluated the clinical impact of the abovementioned rapid ID procedure (15–19). However, the RAPIDO randomized trial revealed that rapid pathogen ID alone did not improve the outcomes of patients with BSIs (20). The authors suggested that the major reason for the lack of improvement in the clinical outcomes of patients is likely a lack of accurate minimum inhibitory concentration (MIC) values of antibiotics, as ID and AST are complementary and interdependent components that together inform and enhance clinical decision-making. Although direct susceptibility test methods from positive blood culture bottles have been developed in recent years (21–24), few studies have evaluated the clinical impact of these rapid blood culture AST methods. Banerjee et al. (25) carried out a prospective randomized controlled trial to evaluate outcomes associated with rapid multiplex PCR (rmPCR) detection of bacteria, fungi, and resistance genes directly from positive blood cultures. Their results revealed that the time from positive blood culture to identification of microorganisms and the time to appropriate antimicrobial de-escalation or escalation were shorter in the rmPCR group than the conventional group. However, the rmPCR group and the conventional group did not differ in terms of mortality, length of hospital stay, or hospital cost (25), findings that were similar to the results of other clinical studies reported by the same authors (10). In this study, the same authors also conducted a multicenter, randomized controlled trial in which the outcomes of patients with gram-negative bacilli bloodstream infections were compared between those who underwent blood culture testing with a conventional culture-dependent process and antimicrobial susceptibility testing and those who underwent rapid organism identification and phenotypic susceptibility testing using the Accelerate Pheno System (11). Rapid organism ID and phenotypic AST decreased the time needed to change antibiotic therapy for gram-negative BSIs, but no difference in patient outcomes was detected between the arms (25). Only one published paper analyzed the impact of a rapid molecular test for *Klebsiella pneumoniae* carbapenemase on outcomes after bacteremia caused by carbapenem-resistant Enterobacterales (CRE) (26). It included 137 patients with CRE bacteremia collected from 2016 to 2018 at eight medical centers in the USA. The results indicated that *bla*KPC PCR testing of positive blood cultures was associated with decreased time until appropriate therapy and decreased mortality for CRE bacteremia. Most of these methods are based on molecular biology techniques. Currently, these methods can provide information that is clinically relevant and complementary but not equivalent to that provided by conventional culture-dependent methods (14). Thus, other innovative approaches and high-level evidence on patient outcomes are still essential for emerging methodologies applied to the early diagnosis of BSIs.

In a previous study, we established a protocol for pathogen ID and AST analysis directly from positive blood culture bottles (27). This protocol provides the MICs of various antibiotics required for clinical use while eliminating the time needed for a second subculture to obtain pure colonies, thus significantly improving the laboratory diagnostic process for bloodstream infections. However, to our knowledge, no studies have evaluated the clinical value of this protocol. Here, we carried out a prospective cohort study to analyze the clinical effect of using the IH rapid test for pathogen ID and AST in BCs positive for CRO or MRSA. The primary outcomes included patient length of stay in the hospital, hospital costs, and 28-day all-cause mortality after BSI. The secondary outcomes included the time until organism identification and susceptibility results and the time to the initiation of effective antibiotic therapy. We aimed to determine whether this laboratory-based rapid diagnosis process could improve clinical outcomes for patients with BSIs due to CRO or MRSA.

## RESULTS

### Patient and baseline data

**Fig 1:**
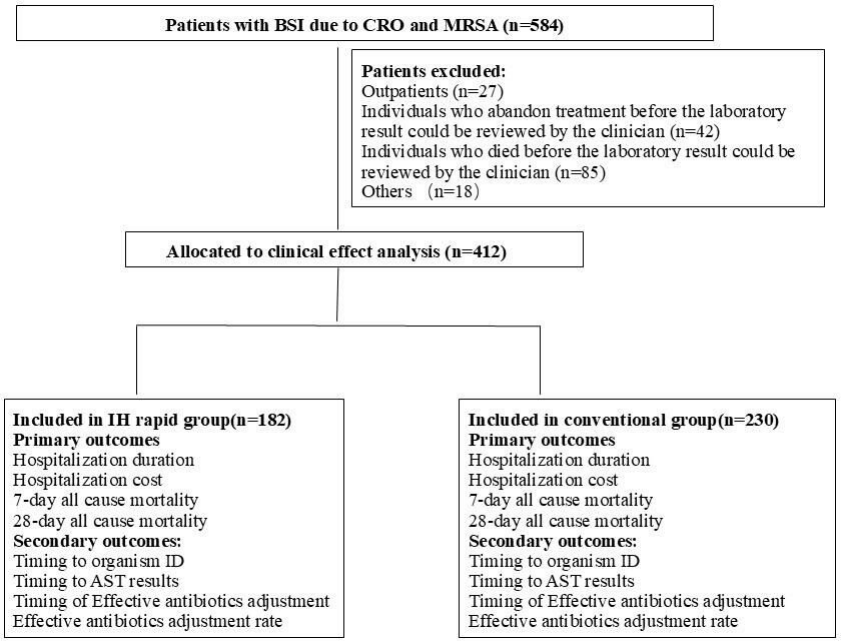
Flowchart of the study participants. AST, antibiotic susceptibility test; BSI, bloodstream infection; ID, identification.

A total of 584 patients with BSIs caused by CRE, CRAB, CRPA, *Stenotrophomonas maltophilia*, *Burkholderia cepacia*, or MRSA were identified between 1 January 2018 and 30 June 2023, and 412 patients were included in the final analysis (Fig. 1). The overall mortality rate of patients due to BSIs with CRO or MRSA in our study was 25.17% (147/584), among whom 14.55% (85/584) died before the laboratory results could be reviewed by a clinician. Thus, these patients were not included in the following analysis.

Among the 412 enrolled patients, the median age was 51 years, and 66.99% were male. The most common comorbidities were hypertension (34.22%), cardiovascular disease (16.50%), and diabetes (14.56%). Intra-abdominal (19.17%), respiratory tract (18.45%), intracranial (16.02%), and vascular (14.08%) catheters were the most common source sites of infections (Table 1). The major CRO pathogens were *K. pneumoniae* (35.44%) and *Acinetobacter baumannii* (27.43%) (Table 1). Among the baseline data, age, Charlson comorbidity score, the presence of cardiovascular disease, admission to the ICU, and infection with CRAB were associated with increased 28-day all-cause mortality after BSIs occurred (Table 1).

**TABLE 1:** Baseline characteristics of the patients with BSIs caused by CRO or MRSA and analysis for predictors of 28-day mortality after BSIs occurred*^a^**^,b^*

Characteristics	Total (*n* = 412)	Survivors (*n* = 350)	Died within 28 days (*n* = 62)	*P* value
Demographics				
Male sex	276 (66.99)	232 (66.29)	44 (70.97)	0.47
Age (years), median (IQR)	51 (31–62)	49 (26.75–61.0)	60 (45.75–69.75)	<0.01
Coexisting conditions				
Charlson comorbidity score, median (IQR)	2 (0–3)	1 (0–3)	2 (1–4)	<0.01
Leukemia and lymphoma	39 (9.47)	34 (9.71)	5 (8.06)	0.68
Tumor	29 (7.04)	22 (6.29)	7 (11.29)	0.16
Diabetes mellitus	60 (14.56)	51 (14.57)	9 (14.52)	0.99
Chronic kidney disease	2 (0.49)	2 (0.57)	0 (0.00)	0.55
Chronic liver disease	13 (3.16)	12 (3.43)	1 (1.61)	0.45
Cardiovascular disease	68 (16.50)	51 (14.57)	17 (27.42)	0.01
Hypertension	141(34.22)	113 (32.29)	28 (45.16)	0.05
Organ transplant	8 (1.94)	7 (2.00)	1 (1.61)	0.84
Enrolled in ICU	100 (24.27)	74 (21.14)	26 (41.94)	<0.01
Source of BSIs				
Catheter related	58 (14.08)	45 (12.86)	13 (20.97)	0.09
Intra-abdominal	79 (19.17)	67 (19.14)	12 (19.35)	0.97
Respiratory	76 (18.45)	59 (16.86)	17 (27.42)	0.05
Skin/soft tissue	37 (8.98)	34 (9.71)	3 (4.84)	0.22
Urinary	15 (3.64)	14 (4.00)	1 (1.61)	0.36
Intracranial infection	66 (16.02)	57 (16.29)	9 (14.52)	0.73
Strains				
CRKP	146 (35.44)	121 (34.57)	25 (40.32)	0.38
CRAB	113 (27.43)	88 (25.14)	25 (40.32)	0.01
Other CRE	39 (9.47)	38 (10.86)	1 (1.61)	0.02
CRPA	27 (6.55)	21 (6.00)	6 (9.68)	0.28
*Stenotrophomonas maltophilia*	19 (4.61)	17 (4.86)	2 (3.23)	0.57
*Burkholderia* spp.	12 (2.91)	11 (3.14)	1 (1.61)	0.51
MRSA	56 (13.59)	54 (15.43)	2 (3.23)	0.01

^*a*^ Data are no. (%) or median with IQR.

^*b*^ AB, *Acinetobacter baumannii*; CR, carbapenem resistant; E, Enterobacteriaceae; ICU, intensive care unit; IQR, interquartile range; KP, *Klebsiella pneumoniae*; MRSA, methicillin-resistant *Staphylococcus aureus*; PA, *Pseudomonas aeruginosa.*

The IH and conventional groups included 182 and 230 patients, respectively. Baseline characteristics, including age and sex distributions, clinical status, comorbidities, and source of BSIs, were similar, and no significant difference was observed between the two randomized groups (Table 2). The CRO pathogens or MRSA responsible for the BSIs in the two groups are listed in Table 3. The bacterial distributions were generally comparable between the two groups. The most common CRO pathogens were *K. pneumoniae* and *A. baumannii* in both groups. However, more patients in the conventional group seemed to have *S. maltophilia* bacteremia (*P* = 0.01) (Table 3).

**TABLE 2:** Demographic and clinical characteristics of patients with BSIs caused by CRO or MRSA in the IH group and conventional group^*a*^^*,*^^*b*^

Characteristic	IH group (*n* = 182)	Conventional group (*n* = 230)	*P* value
Demographics			
Male sex	123 (67.58)	154 (66.96)	0.89
Age (years), median (IQR)	52 (33–63)	50 (27–62)	0.39
Age <18 years	31 (17.03)	48 (20.87)	0.55
Coexisting conditions			
Charlson comorbidity score, median (IQR)	2 (0–3)	2 (0–3)	0.52
APACHE II score (mean ± SD)	21.8 ± 6.4	21.6 ± 6.6	0.53
Leukemia, lymphoma	17 (9.34)	22 (9.57)	0.94
Tumor	13 (7.14)	16 (6.96)	0.94
Diabetes mellitus	28 (15.38)	32 (13.91)	0.67
Chronic kidney disease	0 (0.00)	2 (0.87)	0.21
Chronic liver disease	5 (2.75)	8 (3.48)	0.67
Cardiovascular disease	34 (18.68)	34 (14.78)	0.29
Hypertension	66 (36.26)	75 (32.61)	0.44
Organ transplant	5 (2.75)	3 (1.30)	0.29
Admitted to ICU	48 (26.37)	52 (22.61)	0.38
Source of bloodstream infection			
Catheter related	20 (10.99)	38 (16.52)	0.11
Intra-abdominal	37 (20.33)	42 (18.26)	0.60
Respiratory	33 (18.13)	43 (18.70)	0.88
Skin/soft tissue	18 (9.89)	19 (8.26)	0.57
Urinary	10 (5.49)	5 (2.17)	0.07
Intracranial infection	25 (13.74)	41 (17.83)	0.26
Unidentified	39 (21.43)	42 (18.26)	0.42

^*a*^ APACHE II score only reported for patients admitted to the ICU. Data are no. (%) or mean ± standard deviation or median with (IQR).

^*b*^ APACHE II, Acute Physiologic Assessment Chronic Health Evaluation II; ICU, intensive care unit; IQR, interquartile range; SD, standard deviation.

**TABLE 3:** CRO and MRSA distribution of BSIs^*a*^^*,*^^*b*^

Bacterial organisms		Episodes (*n* = 412)		*P* value
		IH group (*n* = 182)	Conventional group (*n* = 230)	
Enterobacteriaceae bacteria	CRKP	66 (36.26)	80 (34.78)	0.76
	CREco	6 (3.30)	9 (3.91)	0.74
	CREnt	11 (6.04)	7 (3.04)	0.14
	Others	3 (1.65)	3 (1.30)	1.00
CRAB		51 (28.02)	62 (26.96)	0.81
CRPA		8 (4.40)	19 (8.26)	0.12
*Stenotrophomonas maltophilia*		3 (1.65)	16 (6.96)	0.01
*Burkholderia* spp.		6 (3.30)	6 (2.61)	0.68
MRSA		28 (15.38)	28 (12.17)	0.35

^*a*^ Data are given as numbers and percentages.

^*b*^ AB, *Acinetobacter baumannii*; CR, carbapenem resistant; Eco,* Escherichia coli*; Ent, *Enterobacter cloacae*; KP, *Klebsiella pneumoniae*; MRSA, methicillin-resistant *Staphylococcus aureus*; PA, *Pseudomonas aeruginosa*.

### Clinical outcomes

**Fig 2:**
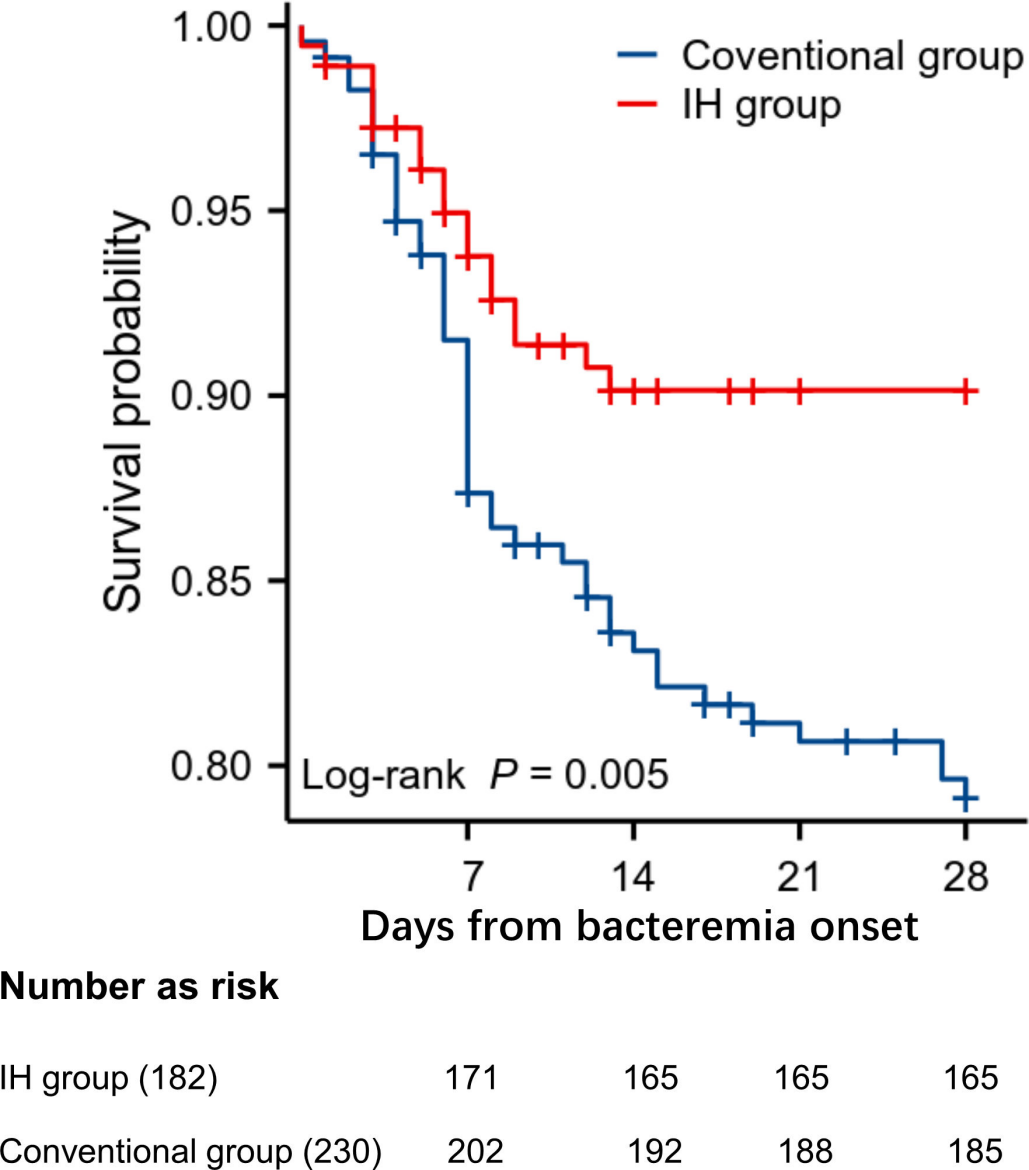
Kaplan‒Meier analysis of overall 28-day survival in both study groups (*P* = 0.005).

The primary outcomes, including 7- and 28-day all-cause mortality after BSIs occurred, hospitalization duration, and hospital costs, are shown in Table 4. In the IH group and conventional group, 6.04% (11/182) and 12.17% (28/230) of the patients, respectively, had died by Day 7, and this difference was significant (*P* = 0.04). Furthermore, a greater overall significant reduction (9.34% vs 19.57%, *P* < 0.01) in 28-day all-cause mortality was observed between the IH group and the conventional group. A Kaplan‒Meier plot of 28-day survival is shown in Fig. 2. Although no significant reduction in length of hospitalization or hospital costs was noted for the IH group, the average cost ($20,423.7 vs $26,515.7; *P* = 0.63) decreased in the rapid test group compared with the conventional group (Table 4).

**TABLE 4:** Clinical and treatment-related outcomes^*a*^^*,*^^*b*^

Outcomes	Total		
	IH group (*n* = 182)	Conventional group (*n* = 230)	*P* value
Primary clinical outcomes			
Length of hospitalization (days), median (IQR)	29 (18–42)	29 (20–44)	0.56
Total cost ($), median (IQR)	20,423.7 (10,119.0–50,461.7)	26,515.7 (12,097.2–44,706.3)	0.63
7-day all-cause mortality	11 (6.04)	28 (12.17)	0.04
28-day all-cause mortality	17 (9.34)	45 (19.57)	0.01
Secondary clinical outcomes			
Turnaround time			
Mean time from blood culture positivity to organism identification (h), median (IQR)	8.58 (4.85–12.88)	31.06 (22.90–36.44)	<0.01
Mean time from blood culture positivity to reporting of susceptibilities (h), median (IQR)	31.82 (28.38–36.44)	55.70 (46.98–62.35)	<0.01
Treatment-related outcomes			
Effective antibiotics adjust rate	128 (70.33)	129 (56.09)	<0.01
Time to effective therapy (h), median (IQR)	34.34 (15.64–51.41)	50.02 (29.70–69.07)	<0.01

^*a*^ IQR, interquartile range.

^*b*^ Data are given as numbers and percentages or medians with IQRs.

The secondary clinical outcomes included the timing to organism ID, the timing to AST results, the timing of effective antibiotic adjustments, and the effective antibiotic adjustment rate (Table 4). Compared with the conventional culture-dependent ID and AST processes, the rapid IH ID and AST process not only significantly decreased the time to pathogen identification but also resulted in a significantly shorter time to providing susceptibility results. The median times from a positive blood culture flag to pathogen identification were 8.58 and 31.06 h for the IH process and subculture-dependent conventional workflow, respectively (*P* < 0.01). The median times from a positive blood culture flag to the susceptibility results being sent to clinicians (AST reporting) were 31.82 and 55.70 h for the rapid test and subculture-dependent workflow, respectively (*P* < 0.01).

**Fig 3:**
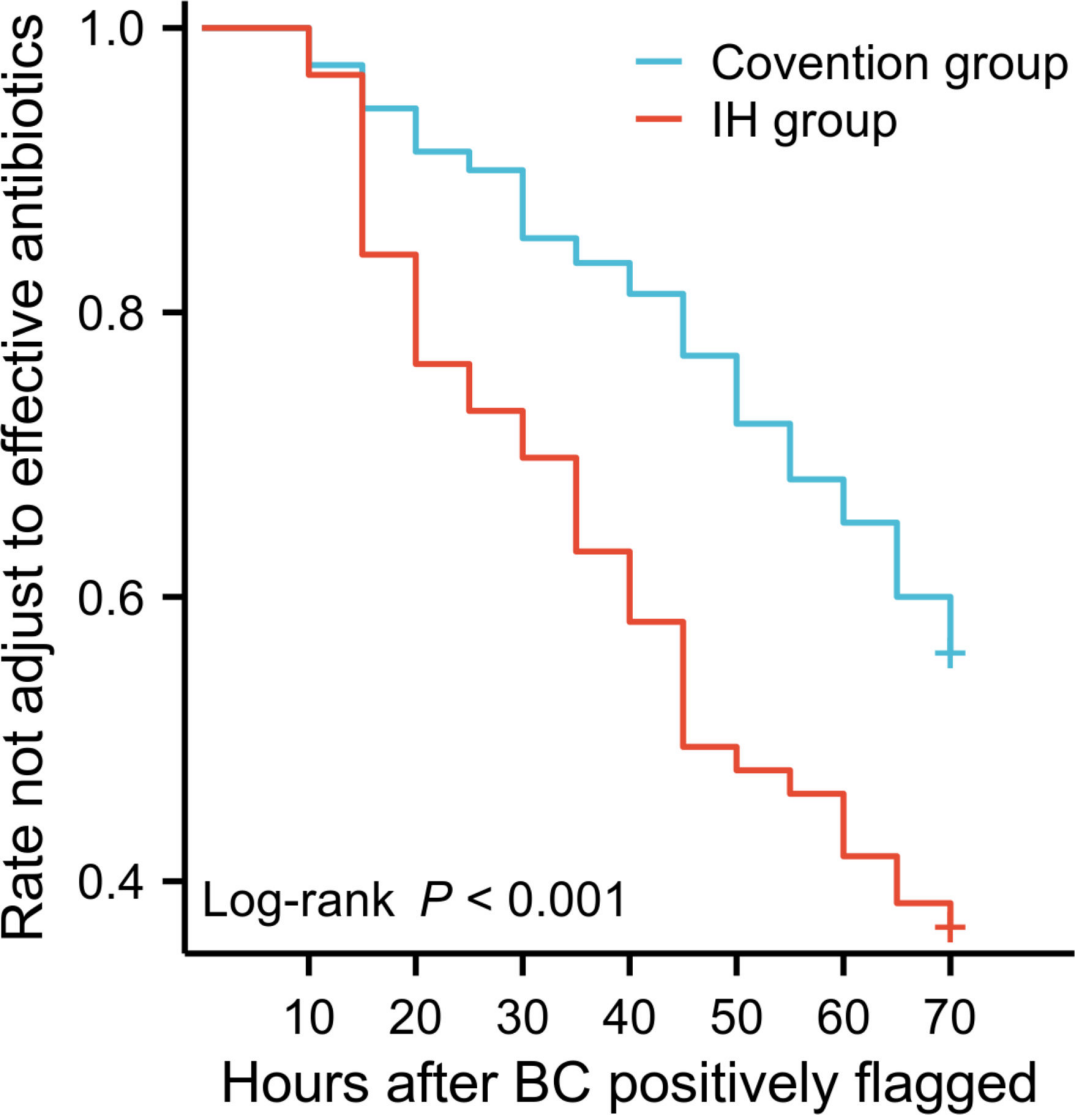
Kaplan‒Meier curve for time to initiation of appropriate antimicrobial therapy. BC, blood culture. *P* < 0.01.

Shortened laboratory reporting times provide early warning of CRO and MRSA to clinicians, which can shorten the time needed to adjust treatment to a more effective antibiotic. Among the 182 BSI patients in the IH group, antimicrobial treatment was adjusted for 128 patients (70.33%), whereas only 129 of the 230 (56.09%) patients in the conventional group had their treatment adjusted (*P* < 0.01). Effective antibiotic adjudication was significantly more common in the IH group than in the conventional group. Moreover, the time from a positively flagged blood culture to effective therapy also decreased from 50.02 to 34.34 h (*P* < 0.01). The Kaplan‒Meier curve for the time to initiation of appropriate antimicrobial adjustments after a blood culture was positive is shown in Fig. 3.

In addition, bivariate and multivariate unconditional logistic regression analyses for predictors related to 28-day mortality were carried out. All sources of infection, coexisting conditions, and types of organisms were included in the bivariate analysis, and all covariates with *P* < 0.05 are listed in Table 5. Consistent with the results listed in Table 1, age, the Charlson comorbidity score, the presence of cardiovascular disease, admission to the ICU, and infection with CRAB were associated with increased 28-day all-cause mortality in the bivariate analysis. Multivariate logistic regression analysis revealed that admission to the ICU and the presence of cardiovascular disease factors were significantly related to 28-day all-cause mortality (Table 5). As no significant difference in these clinical characteristics was observed between the two randomized groups (Tables 2 and 3), survival curve correction was unnecessary in our study. Notably, the IH process was associated with decreased unadjusted odds of 28-day mortality according to multivariate logistic regression analysis results (odds ratio: 0.36, 95% confidence interval [CI]: 0.19–0.68; *P* < 0.01).

**TABLE 5:** Bivariate and multivariate unconditional logistic regression analyses for predicting 28-day mortality^*a*^^,^^*b*^

	Bivariate analysis			Multivariate logistic regression analysis		
	Death (*n* = 62)	No death (*n* = 350)	*P* value	OR	95% CI	*P* value
Coexisting conditions						
Age	60 (45.75–69.75)	49 (26.75–61)	<0.01	1.52	0.66–3.45	0.33
Charlson comorbidity score	2 (1–4)	1 (0–3)	0.01	1.28	0.58–2.86	0.53
Enrolled in ICU	26 (41.94)	74 (21.14)	<0.01	2.30	1.25–4.24	<0.01
Cardiovascular disease	17 (27.42)	51 (14.57)	<0.01	2.24	1.09–4.60	0.03
Source of bloodstream infection						
Respiratory	17 (27.42)	59 (16.96)	0.02	1.48	0.76–2.90	0.25
Pathogens						
CRAB	25 (40.32)	88 (25.14)	0.02	1.50	0.82–2.76	0.19
MRSA	2 (3.23)	54 (15.43)	0.02	0.24	0.05–1.08	0.06

^*a*^ Data are given as numbers and percentages. All sources of infection, coexisting conditions, and types of organisms were included in the bivariate analysis, and all covariates with *P* < 0.05 are listed in the table.

^*b*^ CI, confidence interval.

Overall, our results demonstrated that laboratory-based rapid ID and AST greatly improved clinical outcomes and significantly decreased the mortality of patients with BSIs due to CRO or MRSA. Interestingly, a subgroup analysis for 28-day mortality revealed significant differences in the effects of rapid IH diagnosis for carbapenem-resistant nonfermenting gram-negative bacterial strains (3.85% [7/182] vs 12.17% [28/230], *P* < 0.01). These results suggest that the IH rapid diagnostic test benefits patients with BSIs caused by this type of bacteria.

## DISCUSSION

Septicemia remains a major cause of hospital mortality (14). Laboratory-based timely and accurate reporting of microbiologic data is a critical determinant of clinical outcomes for patients with BSIs. Our research provides further support for this argument, as 14.55% (85/584) of patients with BSIs due to CRO or MRSA died before the laboratory report could be reviewed by the clinician. The results indicate that “waiting for susceptibility” is not a safe default for patients with high-risk bacteremia, especially for bacteremia caused by CRO and MRSA. Early adjustment guided by rapid diagnostics and risk-based protocols is warranted when the prevalence of CRO is high. A variety of rapid diagnostic systems have been developed for the rapid ID of pathogens in BSIs to provide results more quickly than routine methods do (14). However, the RAPIDO randomized trial revealed that rapid pathogen ID alone did not improve the outcomes of patients with BSIs (20). From our point of view, the lack of AST results is the major reason for the lack of improvement in the clinical outcomes of these patients. We believe that accurate MIC values of antibiotics are far more important than pathogen ID in BSIs. Most current direct AST methods from positive blood culture bottles are based on the detection of resistance genotypes through molecular biology techniques (28). This type of detection has certain limitations. First, genetic resistance does not always correlate with phenotypic susceptibility. Second, these tests are limited to facilitating drug avoidance rather than guiding clinicians in choosing the best treatment. Therefore, these methods cannot replace conventional laboratory AST procedures.

In a previous study, we established a protocol for pathogen identification and AST analysis directly from positive blood culture bottles (27). This protocol provides the MIC values of various antibiotics required for clinical use while eliminating the time needed for a second subculture to obtain pure colonies, thus significantly improving the laboratory diagnostic process for bloodstream infections. However, to our knowledge, no studies have evaluated the clinical value of this protocol. Thus, the present study focused on an analysis of the clinical benefits of laboratory-based rapid ID combined with AST of the pathogens causing BSIs. A total of 584 patients were included in the study, and data from 412 of these patients with BSIs due to CRO or MRSA were used for clinical effect analysis. The use of the rapid IH test for ID and AST processes significantly decreased the laboratory reporting time, which improved the time to effective antibiotic therapy and increased the use of effective antibiotics. Thus, 7- and 28-day mortality markedly decreased in the rapid IH group (Table 4; Fig. 2). Our results revealed that rapid AST is necessary for improving the clinical outcomes of patients with BSIs due to CRO or MRSA. Owing to understaffing, we could only process samples during the workday; thus, the entire reporting cycle was slightly longer than that in other studies (10). Although the average costs appeared to decrease in the rapid group compared with those in the conventional group, the difference was not statistically significant. Notably, these results differ from those reported in other studies (19) perhaps because the hospitalization period and expenses were not attributed to BSIs alone, as most patients enrolled in our study often experienced complications. Moreover, even after the final report was issued, fewer adjustments of antibiotics were made in the conventional group than in the IH group. One likely reason for these findings is that the patients had already been receiving appropriate antibiotics. Another key factor is that the AST results of the IH group were obtained within 36 h, when the patient’s condition was often still unstable, making adjustments more acceptable. In contrast, the conventional arm usually received AST reports after 50 h; by that time, some patients had already stabilized on clinical therapy, leading clinicians to perceive ongoing improvement and abstain from further antimicrobial adjustment.

Our research has several advantages over previous studies. First, we selected patients with BSIs due to CRO or MRSA for the clinical effect analysis. In the initial study design, all patients with any initial positive blood culture from our microbiology laboratory were consecutively screened and prospectively enrolled for analysis regardless of the suspected pathogen. However, the results revealed no significant difference in clinical outcomes between the IH group and the conventional group. These results were consistent with those of randomized controlled trials conducted by Banerjee et al. (25). Clinicians usually empirically prescribe typical broad-spectrum antibiotics to ensure that all potential pathogens are targeted and decrease the high mortality risk of BSIs (29). These broad-spectrum antibiotics are often effective against sensitive bacterial strains responsible for BSIs. In this case, clinicians may continue the current treatment strategies. Therefore, objectively evaluating the clinical effect of a laboratory-based rapid test to identify and obtain AST results of pathogens that cause BSIs is difficult. This difficulty may be one of the key factors contributing to the lack of clinical differences in the two randomized controlled trials conducted by Banerjee et al. (25). Empirical treatment often fails to cover multidrug-resistant bacterial strains, such as CRO and MRSA, which cause higher mortality than sensitive strains do. Thus, only patients with BSIs due to CRO or MRSA were included in our final analysis to objectively evaluate the clinical effect of a laboratory-based rapid test to identify the involved pathogens and obtain AST results. Second, the study covered all BSI inpatients infected with CRO or MRSA from 1 January 2018 to 30 June 2023. Patients were allocated to the IH or conventional group at random, which can exclude confounding factors and reflect the most real clinical effect. Third, rapid pathogen ID combined with AST was employed in our study; thus, we provided clinicians with the most accurate antimicrobial MIC values 23.88 h earlier than conventional methods (Table 4). To the best of our knowledge, no study has employed such a procedure to analyze clinical effects. Therefore, we believe that our research findings will encourage the implementation of laboratory-based rapid diagnosis of BSIs.

Although the research was conducted at a single center, a relatively large group of patients (412) with BSIs due to CRO or MRSA were enrolled in the study. The only previous study included only 137 patients with CRE bacteremia collected from 2016 to 2018 from eight medical centers in the USA (26). Thus, to some extent, the reliability of the conclusions can be ensured. Because of the clinical complications of most patients enrolled, analyzing the clinical benefits of the rapid IH test for patients with only BSIs was difficult. We believe that the clinical benefits will be more significant if the rapid IH process is employed only for the diagnosis and treatment of patients with BSIs.

In conclusion, our laboratory-based rapid ID and AST process of positive BCs significantly shortened laboratory reporting times, which led to improvements in the rate and timing of the delivery of effective antibiotic therapy and a significant decrease in the mortality of patients with BSIs due to CRO or MRSA. This information encourages the implementation of laboratory-based rapid diagnosis for BSIs.

## MATERIALS AND METHODS

### Study design and patients

We conducted a prospective, single-center cohort study of all patients with BSIs caused by CRO (including *Enterobacteriaceae* [CRE], *Acinetobacter baumannii* [CRAB], *Pseudomonas aeruginosa* [CRPA], *Stenotrophomonas maltophilia*, and *Burkholderia cepacia*) and MRSA. This study was conducted at Qilu Hospital of Shandong University, a national-level regional medical center in China with more than 4,000 beds and two intensive care units. The hospital admits approximately 187,600 inpatients annually. This study was performed by the Division of Clinical Microbiology in the Department of Clinical Laboratory.

All patients admitted to the hospital from 1 January 2018 to 30 June 2023, for whom at least one blood culture was positive for CRE, CRAB, CRPA, *S. maltophilia*, *B. cepacia*, or MRSA, were included in the study. Individuals who discontinued treatment or who died before the laboratory results could be reviewed by clinicians were excluded because an infectious disease consultation could not reasonably have been obtained. Outpatients whose clinical details could not be verified were also excluded from the study. Individuals whose information was incomplete were also excluded from the study. In the end, 412 of 584 patients with BSIs due to CRO or MRSA were included (Fig. 1).

The clinical effect of using the IH rapid process to diagnose BSIs due to CRO or MRSA was analyzed. A total of 412 patients with positive BCs due to CRO or MRSA were assigned to one of the following arms: (i) IH rapid ID and AST (IH group, 182 patients) vs (ii) conventional culture-dependent process (conventional group, 230 patients) (27). Patients whose blood culture was positive between 18:00 and 10:00 on weekdays were assigned to the IH group, and patients whose blood culture was positive between 11:00 and 18:00 and holidays were assigned to the conventional group. The primary outcomes included a patient’s length of stay in the hospital, hospital costs, and 7- and 28-day all-cause mortality after BSIs occurred. Here, hospital cost refers to the total expenses incurred during the entire hospital stay, i.e., from the time the patient is admitted to the time of discharge. The secondary outcomes were the measurement time until organism identification, susceptibility results, time to the initiation of effective antibiotic therapy, and effective antibiotic adjustment rates (Fig. 1).

### Procedures

Blood cultures were analyzed for the presence of microorganisms using the Bactec plus/F system (Becton Dickinson, Franklin Lakes, NJ, USA). The empiric and directed treatment regimens for bloodstream infections were implemented by the attending physicians with reference to the *Sanford Guide to Antimicrobial Therapy*. The attending physicians were blinded to the randomization. The conventional culture-dependent processes for the ID and AST of microorganisms from positive BCs were performed as previously described, including MALDI-TOF-based identification and resistance testing of colonies grown as subcultures (30). The rapid IH process was carried out as previously reported (27). In this process, MALDI-TOF-based ID and AST were carried out directly from the positive BCs, eliminating the time needed for a second subculture to obtain pure colonies. Organism resistance was tested using the Vitek II system (AES software; bioMérieux, Marcy l’Étoile, France). The detailed protocols for the conventional and IH rapid processes are listed in the supplementary files. The ID and AST results were communicated directly to the clinical teams, who advised on further patient management during both study periods (Fig. 1). Antimicrobial therapy was considered effective if the organism was classified as susceptible to the prescribed antibiotic.

The Qilu Hospital Healthcare Medical Informatics database was used to retrieve relevant clinical information about the enrolled patients. The primary outcomes were patient length of stay, hospital costs, and 7- and 28-day all-cause mortality after BSIs occurred. The secondary outcomes were the time until organism ID and AST results were obtained and the time to administration of effective antibiotic therapy. The time at which a BC was flagged as positive was designated as the starting point for the ID and AST. The time to optimal therapy was defined as the interval from when the BCs were flagged to the administration of effective antimicrobial therapy based on patient-specific susceptibility. Adjustments were made from the moment the first positive blood culture was flagged until 72 h after the final phenotypic AST report.

### Statistical analysis

Student’s *t*-test was employed for the analysis and comparison of normally distributed variables, and the Mann‒Whitney *U* test was used for nonnormally distributed variables. Pearson’s χ^2^ test or Fisher’s test was employed to compare variables between the two baseline categorical arms, if applicable. The results are presented as medians with interquartile ranges or means ± standard deviations or as percentages of the group, if applicable. Twenty-eight-day all-cause mortality was evaluated using the Kaplan‒Meier method and compared using the log-rank test. Time-to-event antimicrobial-related data were also evaluated using the Kaplan‒Meier method and compared using the log-rank test. Odds ratios and 95% CIs were calculated for mortality associations that emerged at *P* < 0.05. All tests were two tailed, and a *P* value of <0.05 was deemed a priori to represent statistical significance. Statistical analyses were carried out using SPSS v.26.0 (Windows, Chicago, USA).

## SUPPLEMENTAL MATERIAL

Supplemental material spectrum.01746-25-s0001.docx Additional experimental details; Fig. S1.

ASM does not own the copyrights to Supplemental Material that may be linked to, or accessed through, an article. The authors have granted ASM a non-exclusive, world-wide license to publish the Supplemental Material files. Please contact the corresponding author directly for reuse.
