# Supplementary material for: Clinical effect of in-house rapid diagnostic process on patients with bloodstream infections due to carbapenem-resistant bacteria or methicillin-resistant Staphylococcus aureus: a prospective cohort study
Source: Microbiol Spectr. 2026 Jan 8;14(2):e01746-25. doi: 10.1128/spectrum.01746-25 (PMC12889151; doi:10.1128/spectrum.01746-25)
Supplement: Supplemental material — Additional experimental details; Fig. S1. [file spectrum.01746-25-s0001.docx]

**Supplementary procedure and Figure**

**Conventional culture-dependent blood culture processing**

All bacteria-positive bottles were subjected to routine laboratory culture to obtain colonies for MALDI-TOF MS ID and AST as previously described^20^.

**IH blood culture processing**

Positive blood culture specimens were processed using our developed IH method (27). Briefly, a 1-mL aliquot from a positive blood culture was transferred to a 15-mL tube, 3 mL of lysis buffer added, and the final solution incubated at room temperature until the blood became transparent. The lysis buffer contained 1 g/L KHCO_3_, 8.3 g /L NH_4_Cl, and 0.037 g /L EDTA-Na_2_. The bacterial cells were pelleted by centrifugation (3,500 × g for 5 min) and resuspended in 1 mL of 0.45 % saline solution for washing. The bacterial suspension was pelleted after centrifugation (10,000 × g for 2 min) and the supernatant was discarded. The resulting pellet was resuspended in 0.45 % saline solution. The bacterial suspension was then split into two 1.5-mL Eppendorf tubes for direct MALDI-TOF MS (Bruker Daltonics) identification and Vitek 2 (bioMérieux) AST, respectively. The bacterial cell pellet was dissolved by vortexing in 50 μL of 70 % formic acid. Next, 50 μL of pure acetonitrile were added to the solution and vortexed, and samples were centrifuged (10,000 × g for 2 min) prior to direct identification by MALDI-TOF MS (Bruker Daltonics).

**MALDI-TOF MS analysis**

MALDI-TOF MS identification was carried out as previously described (27). One microliter of the supernatant was spotted onto a steel target plate, air-dried and immediately overlaid with 1 μL of HCCA matrix solution (Bruker Daltonics). Microorganism identification was performed by using the Bruker microflex MALDI-TOF MS system and the MALDI Biotyper 3.0 RTC database (Bruker Daltonics). The database used for bacterial identification was MALDI Biotyper 5989.

**AST analysis**

AST was carried out as described above. In brief, the cell density of the remaining bacterial suspension was adjusted to a density of 0.5 McFarland after dilution in 0.45 % saline; 145 µL of the bacterial suspension was drawn into 3 mL of 0.45 % saline solution to adjust further the bacterial cell density. Vitek cards were inoculated with the suspension vials and loaded into the Vitek 2 automated reader-incubator. Vitek cards AST-GN13, AST-GN16, AST-GN09, AST-GN334 and AST-GN335 were used for Gram-negative bacteria; AST-Gp67 and AST-Gp639 were used for Gram-positive bacteria.

**IH rapid methods show high concordance with conventional methods for both bacterial identification and AST.**

As previously reported (27), IH rapid methods demonstrate high concordance with conventional workflows for both bacterial identification and AST. Direct MALDI-TOF MS correctly identified 96.49 % of Gram-negative and 97.22 % of Gram-positive organisms. Direct Vitek-2 AST of Gram-negative isolates yielded 96.89 % categorical agreement with the conventional method, accompanied by 2.63 % minor, 0.24 % major, and 0.24 % very major discrepancies. For Gram-positive isolates, categorical agreement was 92.81 %, with 4.51 % minor, 1.22 % major, and 1.46 % very major errors. Notably, meropenem, imipenem, ertapenem, and oxacillin achieved 100 % categorical agreement, confirming the reliability of the IH protocol for detecting CRO and MRSA.

**
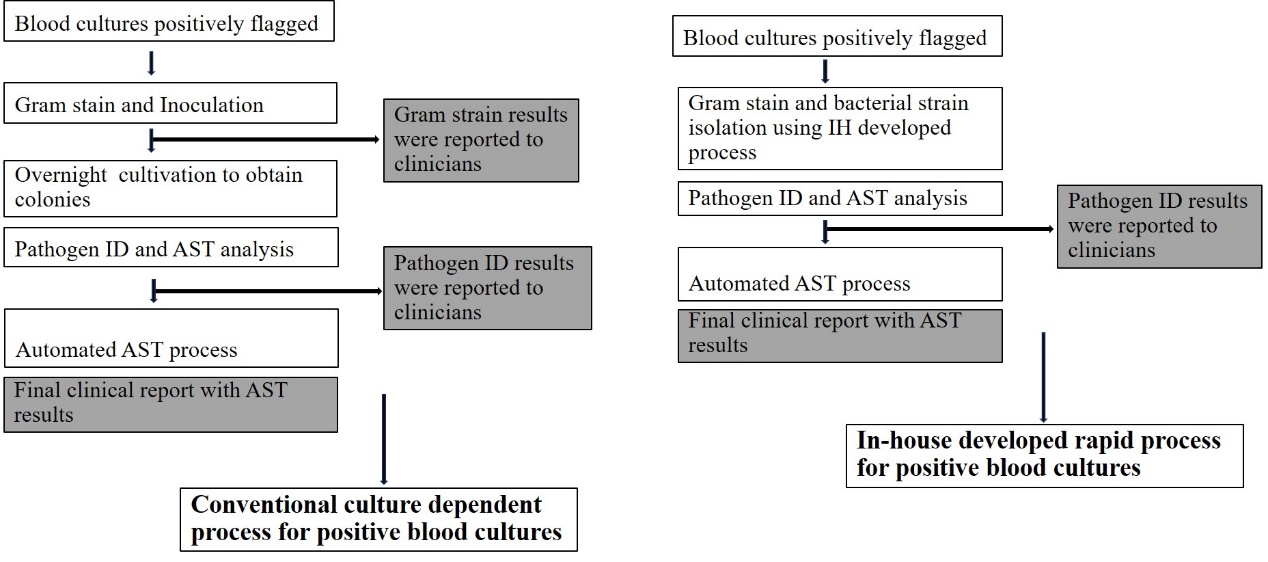
**

**Fig. S1.** **The laboratory report protocols of conventional culture dependent process and the in-house developed rapid process for positive blood cultures.**
